# The relationship between parental feeding practices and children’s health-related quality of life

**DOI:** 10.3389/fnut.2026.1858474

**Published:** 2026-09-03

**Authors:** Charlotte Juton, Silvia Torres, Gina Valdés-Querol, Luis Rajmil, Montserrat Fito, Santiago F. Gómez, Helmut Schröder

**Affiliations:** 1 Department of Health Information, Food Systems, and Nutrition, Sciensano, Brussels, Belgium; 2 Gasol Foundation Europe, Sant Boi de Llobregat, Barcelona, Spain; 3 Department of Health Information, Food systems and Nutrition, Sciensano, Faculty of Health Sciences and Welfare, Research Group on Methodology, Methods, Models and Outcomes of Health and Social Sciences (M3O), Center for Health and Social Care Research (CESS), University of Vic-Central University of Catalonia (UVic-UCC), Vic, Barcelona, Spain; 4 Pediatric and Public Health Specialist, Barcelona, Spain; 5 Cardiovascular Risk and Nutrition Research Group, Hospital del Mar Research Institute, Barcelona, Spain; 6 CIBER Fisiopatología Obesidad y Nutrición (CIBERobn), Instituto de Salud Carlos III, Madrid, Spain; 7 Nursing and Physiotherapy Department, University of Lleida, Lleida, Spain; 8 CIBER Epidemiology and Public Health (CIBERESP), Instituto de Salud Carlos III, Madrid, Spain; 9 Research Institute of Biomedical and Health Sciences, University of Las Palmas de Gran Canaria, Las Palmas de Gran Canaria, Spain

**Keywords:** parental feeding practices, health-related quality of life, restriction, pressure to eat, monitoring, Child Feeding Questionnaire, KIDSCREEN-10

## Abstract

**Background:**

Healthy eating is essential throughout childhood and adolescence, and has been associated with better health-related quality of life (HRQoL). Healthy eating results from complex interactions, including the transmission of eating habits within families. Although parental feeding practices may influence children’s HRQoL through several plausible behavioral pathways, empirical evidence examining this association remains limited. Therefore, the objective of this study was to investigate the association between parental feeding practices—monitoring, pressure-to-eat, and restriction—and children’s HRQoL.

**Methods:**

This cross-sectional study included 1,535 children (819 boys and 716 girls) aged 3 to 11 years recruited from 19 schools in Catalonia between 2022 and 2024. Parental feeding practices and children’s HRQoL were assessed by the same parent using the Child Feeding Questionnaire and the KIDSCREEN-10 parent-proxy instrument, respectively.

**Results:**

Restrictive and pressure-to-eat feeding practices were associated with lower HRQoL in children (*p* < 0.001 and *p* = 0.03, respectively), whereas monitoring showed a significant non-linear association with HRQoL (p < 0.001), with higher HRQoL observed only at the highest levels of monitoring, after adjustment for children’s age, gender, body mass index, adherence to the Mediterranean diet, physical activity level and parental education level.

**Conclusion:**

Based on reports of both parental feeding practices and children’s HRQoL provided by the same parent, our findings were broadly consistent with our hypothesis; however, future longitudinal studies incorporating multiple informants are needed to clarify the directionality of these associations and minimize potential reporting bias.

## 1 Introduction

By measuring well-being in its entirety, taking into account physical, mental and social aspects, health-related quality of life (HRQoL) is a variable of choice for measuring individual well-being (1). Healthy eating is essential throughout childhood and adolescence, and has been associated with better HRQoL (2, 3). However, healthy eating results from complex interactions involving not only the consumption of nutritious foods, but also the social and cultural transmission of eating habits throughout a child’s development, particularly within families. Children’s eating habits are influenced by the way parents manage daily family meals (4–7), and this management may in turn be associated with children’s HRQoL.

Parenting style theory provides a useful conceptual framework for understanding these associations. Baumrind (8) originally described three parenting styles—authoritative, authoritarian, and permissive—with neglectful parenting later added by Maccoby and Martin. Within this framework, parental feeding practices can be viewed as behavioral expressions of broader parenting styles. Indeed, feeding practices explain a proportion of the variance in authoritative and authoritarian parenting, and the implementation of specific feeding practices is related to parenting style (9, 10).

Behavioral self-regulation may represent a potential pathway linking parental feeding practices with children’s HRQoL. Responsive and structured feeding practices (11) may foster children’s ability to recognize and respond appropriately to internal hunger and satiety cues, thereby supporting eating self-regulation (12, 13). Better eating self-regulation may subsequently contribute to healthier dietary behaviors and ultimately better HRQoL. Conversely, coercive control feeding practices (11) may disrupt children’s eating self-regulation, promote less favorable eating behaviors (6, 14) and create negative emotional experiences around food and mealtimes (15), potentially impairing HRQoL.

Monitoring and responsive feeding approaches are more compatible with an authoritative parenting style (9, 10). By monitoring children’s consumption of unhealthy foods, parents may encourage healthier eating habits while allowing children to progressively develop autonomy in their food choices. Together with the direct benefits of a nutritious diet on cognition and emotion (16), these characteristics may support self-control (17) and eating self-regulation, both of which may be associated with better HRQoL.

Conversely, restrictive and pressure-to-eat practices are more aligned with an authoritarian parenting style (9, 10). Restrictive feeding practices, such as limiting access to certain foods or imposing dietary restrictions, may be associated with poorer self-control and regulation (18), poorer body image (19) and lower self-esteem in children. Likewise, pressure-to-eat has been associated with negative emotional experiences around mealtimes and eating (15), which may in turn be associated with poorer physical, emotional, and social well-being and consequently lower HRQoL. Despite these plausible theoretical pathways, evidence examining the association between parental feeding practices and children’s HRQoL remains limited. Therefore, the objective of this study was to investigate the associations between parental feeding practices—including monitoring, pressure-to-eat, and restriction—and children’s HRQoL.

## 2 Methods

### 2.1 Study design and participants

The study was conducted in Sant Boi de Llobregat, located in the metropolitan area of Barcelona, Spain. The Gasol Foundation facilitated the inclusion of the PERCEPS study indicators in the data collection of the SANTBOISÀ cohort study. The current analysis used the baseline cross-sectional data gathered from 19 schools in Catalonia between 2022 and 2024. After removing participants with missing values, a final sample size of 1,535 children (716 girls and 819 boys) aged 3 to 11 years was obtained. The study protocol was approved by the ethics committee of the Fundació Sant Joan de Déu (PIC-144-18), Barcelona, Spain. Parents provided written consent on behalf of their children.

### 2.2 Measures

#### 2.2.1 Parental feeding practices

Parental feeding practices were assessed using the Spanish adaptation of the Child Feeding Questionnaire (CFQ), which measures three dimensions of food parenting: restriction, pressure-to-eat, and monitoring (20). The Spanish version has been validated in parents of Spanish schoolchildren and demonstrated good psychometric properties, including satisfactory internal consistency and factorial validity confirmed by confirmatory factor analysis (21). In the present study, internal consistency was good, with Cronbach’s *α* coefficients of 0.82 for restriction, 0.81 for pressure-to-eat, and 0.89 for monitoring. Restrictive feeding practices, which involve severely restricting unhealthy foods or resorting to instrumental feeding, were assessed using eight specific questions: (1) I have to be sure that my child does not eat too many sweets (candy, ice-cream, cake or pastries); (2) I have to be sure that my child does not eat too many high-fat foods; (3) I have to be sure that my child does not eat too much of her favorite foods; (4) I intentionally keep some foods out of my child’s reach; (5) I offer sweets (candy, ice-cream, cake, pastries) to my child as a reward for good behavior; (6) I offer my child her favorite foods in exchange for good behavior; (7) If I did not guide or regulate my child’s eating, she would eat too many junk foods; (8) If I did not guide or regulate my child’s eating, she would eat too much of her favorite foods. Four questions were used to assess pressure-to-eat feeding practices, which are defined as a tendency to unnecessarily encourage children to eat larger quantities of food: (1) My child should always eat all of the food on her plate; (2) I have to be especially careful to make sure my child eats enough; (3) If my child says “I am not hungry,” I try to get her to eat anyway; (4) If I did not guide or regulate my child’s eating, she would eat much less than she should. Finally, three questions were used to assess monitoring feeding practices, which involve keeping track of the food eaten by children to avoid excessive consumption of unhealthy foods: (1) How much do you keep track of the sweets (candy, ice cream, cake, pies, pastries) that your child eats?; (2) How much do you keep track of the snack food (potato chips, Doritos, cheese puffs) that your child eats?; (3) How much do you keep track of the high fat foods that your child eats?. A 5-point Likert scale, “disagree, slightly disagree, neutral, slightly agree, agree,” was used for the items related to restriction and pressure-to-eat while for monitoring items were rated as follows: “never, rarely, sometimes, mostly, always.” Mean scores were derived by summing the scores assigned to each item within a subscale, where responses were given values ranging from 1 for “disagree” or “never,” up to 5 for “agree” or “always.”

#### 2.2.2 Heath-related quality of life

Children’s HRQoL was assessed using the parent-proxy version of the KIDSCREEN-10 questionnaire, which comprises 10 items assessing children’s perceived health and well-being. The KIDSCREEN-10 parent-proxy version was validated in a multinational sample of 22,830 children and adolescents aged 8 to 18 years and their parents, demonstrating acceptable internal consistency (Cronbach’s *α* = 0.78), strong criterion validity against the KIDSCREEN-52 general HRQoL score (*r* = 0.91), and satisfactory construct validity based on expected correlations with other validated HRQoL instruments (22). In the present sample, the internal consistency of the KIDSCREEN-10 was acceptable (Cronbach’s *α* = 0.74), with similar values observed in children younger than 8 years (*α* = 0.75) and those aged 8 years or older (*α* = 0.74). Parents or caregivers responded to each question using a 5-point Likert scale (1 = “Not at all or never,” 2 = “Slightly or seldom,” 3 = “moderately or quite often,” 4 = “very or very often” and 5 = “extremely or always”). The result was expressed as a total global score ranging from 32.9 to 83.8. Following the KIDSCREEN-10 manual, a Rasch analysis was conducted to include the items that represent a one-dimensional global latent trait of HRQoL. The KIDSCREEN-10 includes questions about feeling fit and well, having energy, experiencing sadness or loneliness, having enough personal time, engaging in desired activities during free time, fair treatment from parents, enjoyment with friends, academic performance, and ability to pay attention.

#### 2.2.3 Adherence to the Mediterranean diet

Adherence to the Mediterranean diet was assessed by the KidMed index (23). This 16-item questionnaire provides a total score ranging from −4 to +12 with higher scores indicating greater adherence to the Mediterranean diet.

#### 2.2.4 Physical activity

Physical activity was evaluated by the physical activity unit 7 item screener (PAU-7S), which comprises 7 questions (24) from which the average daily minutes of moderate-to-vigorous physical activity (MVPA) were calculated.

#### 2.2.5 Anthropometrical variables

Children’s body mass index (BMI) was calculated as kg/m^2^ on the basis of weight and height collected by trained field workers. Weight and height were measured with the subject in underwear and without shoes using an electronic scale (SECA 813, Hamburg, Germany) to the nearest 100 g and a portable stadiometer (SECA 213; Hamburg, Germany) to the nearest 1 mm, respectively.

#### 2.2.6 Parental education

Parental education was defined according to the mother’s highest educational level and categorized into two groups: (1) with university degree and (2) without university degree.

### 2.3 Statistical analysis

Interactions between gender and parental feeding practices were tested to determine whether the analysis could be carried out on the whole sample, or whether it should be stratified accordingly. General linear analysis was performed to investigate the association between the tertiles distribution of parental feeding practices and children’s HRQoL. Three different models (model 1, model 2, and model 3) were used to adjust the analysis for several variables: model 1 was adjusted for gender and age; model 2 was additionally adjusted for child BMI, adherence to the Mediterranean diet and parental education; and model 3 was further adjusted for child physical activity. A general linear analysis divided by age strata was performed to determine whether there was a difference between children aged under 8 and children aged 8 or over, given that the KIDSCREEN-10 validation includes children aged 8 or over. Finally, because categorization may result in some loss of information, we additionally examined continuous dose–response associations using restricted cubic spline analyses between parental feeding practices and children’s HRQoL with the use of the “gam” package in R version 3.0.2. Associations were considered significant if *p* < 0.05. All statistical analyses, with the exception of dose–response analysis, were performed using R (v4.5.2; R Core Team, 2025).

## 3 Results

No interaction between sex and parental feeding practices was found (*p* > 0.05). Table 1 presents the general characteristics of the sample. Table 2 shows the results of the cross-sectional analysis examining the associations between the different types of parental feeding practices (restrictive, pressure-to-eat and monitoring) and children’s HRQoL. According to the general linear analysis (Table 2), higher levels of restriction were associated with lower HRQoL scores in children between the first and second or third tertiles with a significant linear trend in all models. Similarly, higher levels of pressure-to-eat were significantly associated with lower HRQoL particularly between the third and first or second tertiles with a significant trend in all models. Finally, Table 2 showed that higher levels of parental monitoring were associated with higher HRQoL scores between the third and first or second tertiles with a significant linear trend in all models. In the fully adjusted categorical analysis, HRQoL scores were 2.1 and 1.5 points lower in the highest versus lowest tertiles of restriction and pressure-to-eat, respectively, and 2.3 points higher in the highest versus lowest tertile of monitoring. These differences represented approximately 0.15–0.24 standard deviations and were modest in magnitude. Sensitivity analysis (Table 3) revealed similar directions in children aged under 8 years although significance was lost for pressure-to-eat feeding practices. For children aged 8 years or older, the associations between restriction and monitoring and children’s HRQoL were in the same direction as those observed in the overall sample but did not reach statistical significance.

**Table 1:** Characteristics of the study population (*n* = 1,535).

Characteristic	Value
Age (years)	6.5 ± 1.8
Boys (%)	53.4 (819)
Body mass index (kg/m^2^)	16.8 ± 2.4
Adherence to Mediterranean diet (unit)	7.6 ± 2.0
Health-related quality of life (unit)	55.2 ± 9.7
MVPA (min/d)	91.7 ± 44.1
Education (%)	41 (630)
Feeding practices (units)	
Restriction	2.7 ± 1.0
Pressure-to-eat	2.5 ± 1.2
Monitoring	3.9 ± 1.0

Continuous and categorical variables are expressed as mean (standard deviation) and proportions (*n*).

**Table 2:** Cross-sectional association between parental feeding practice and child health related quality of life (HRQoL).

HRQoL^a^	Parental feeding practices tertiles			
	First tertile	Second tertile	Third tertile	*p*-linear trend
Restriction				
	*N* = 515	*N* = 523	*N* = 497	
Model 1	56.7 (55.9 to 57.5)	54.6 (53.8 to 55.4)	54.4 (53.5 to 55.2)	<0.001
Model 2	56.7 (55.8 to 57.5)	54.6 (53.8 to 55.4)	54.4 (53.5 to 55.2)	<0.001
Model 3	56.6 (55.8 to 57.4)	54.6 (53.7 to 55.4)	54.5 (53.7 to 55.3)	<0.001
Pressure-to-eat				
	*N* = 576	*N* = 488	*N* = 471	
Model 1	55.7 (54.9 to 56.5)	55.7 (54.9 to 56.6)	54.1 (53.3 to 55.0)	0.01
Model 2	55.7 (54.9 to 56.5)	55.8 (54.9 to 56.6)	54.1 (53.2 to 55.0)	<0.01
Model 3	55.7 (54.9 to 56.5)	55.7 (54.8 to 56.5)	54.2 (53.3 to 55.1)	0.03
Monitoring				
	*N* = 617	*N* = 416	*N* = 502	
Model 1	54.6 (53.9 to 55.4)	53.8 (52.9 to 54.7)	57.1 (56.3 to 58.0)	<0.001
Model 2	54.7 (53.9 to 55.4)	53.8 (52.9 to 54.7)	57.1 (56.3 to 57.9)	<0.001
Model 3	54.7 (54.0 to 55.5)	53.8 (52.9 to 54.7)	57.0 (56.2 to 57.9)	<0.001

General linear models were fitted to analyze the cross-sectional association between parental feeding practices with child HRQoL. Values are expressed as means (confidence intervals). Different letters indicate significant differences. Model 1 was adjusted for gender and age. Model 2 was adjusted for co-variables in model 1 and, additionally, for child BMI, adherence to Mediterranean diet and parental education. Model 3 was adjusted for co-variables in model 2 and, additionally, for child physical activity.

a HRQoL in children ranged from 32.9 to 83.8.

**Table 3:** Cross-sectional association between parental feeding practice and child HRQoL per age group.

HRQoL^a^	Parental feeding practices tertiles						
	First tertile		Second tertile		Third tertile		*p*-linear trend
Restriction							
	*N*		*N*		*N*		
≤7.99	383	56.6 (55.6 to 57.6)	397	54.4 (53.4 to 55.4)	368	54.7 (53.7 to 55.8)	0.003
≥8.00	131	55.4 (51.7 to 59.1)	125	54.0 (50.2 to 57.8)	127	52.7 (48.7 to 56.6)	0.07
Pressure-to-eat							
	*N*		*N*		*N*	*N* = 355/114	
≤7.99	433	55.6 (54.6 to 56.6)	360	55.7 (54.6 to 56.7)	355	54.5 (53.4 to 55.6)	0.21
≥8.00	143	54.7 (51.0 to 58.4)	126	54.9 (51.2 to 58.6)	114	52.3 (48.3 to 56.3)	0.05
Monitoring							
	*N*		*N*		*N*		
≤7.99	444	54.5 (53.5 to 55.4)	308	54.0 (52.8 to 55.1)	396	57.4 (56.4 to 58.4)	<0.001
≥8.00	173	54.6 (50.9 to 58.3)	106	52.6 (48.6 to 56.6)	104	54.9 (51.1 to 58.7)	0.13

General linear models were fitted to analyze the cross-sectional association between parental feeding practices with child HRQoL. Values are expressed as means (confidence intervals). Different letters indicate significant differences. The model was adjusted for gender, child age, child BMI, adherence to Mediterranean diet, parental education and for child physical activity.

a HRQoL in children ranged from 32.9 to 83.8.

**Figure 1:**
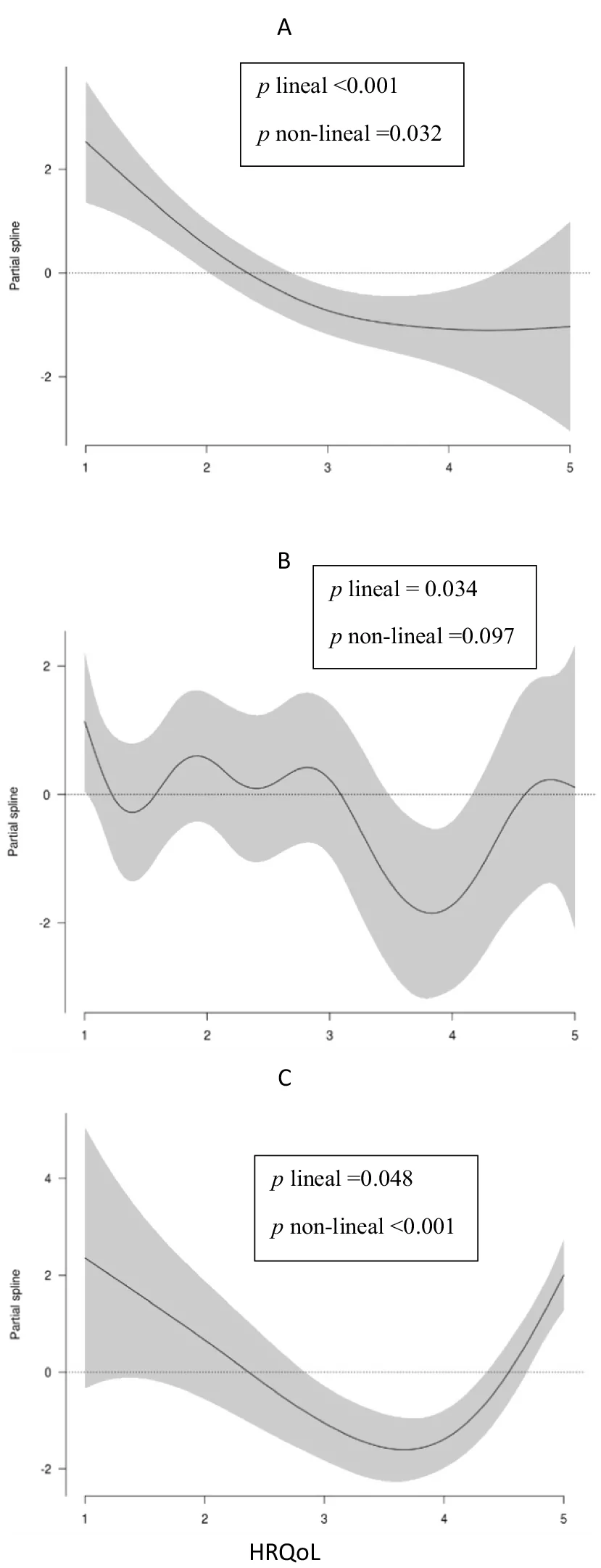
Dose response analysis between parental feeding practices and child health related quality of life (HRQoL). **(A)** Restrictive; **(B)** Pressure-to-eat, **(C)** Monitoring. Models were adjusted for child age, gender, BMI, adherence to Mediterranean diet, physical activity, and parental education. The gray shaded areas represent the 95% confidence bands.

Figure 1 presents the dose–response analyses between restriction (A), pressure-to-eat (B), and monitoring (C) and children’s HRQoL. Models were adjusted for age, sex, parental education, child BMI, physical activity, and adherence to the Mediterranean diet. For restriction, the linear association was significant [*β* = −1.039 (95%CI −1.519; −0.561), *p* < 0.001], and the significant non-linear component (*p* = 0.032) indicated that the relationship was not purely linear. Specifically, HRQoL decreased progressively with increasing levels of parental restriction, with the decline being steeper at lower restriction scores and gradually leveling off at higher levels of restriction. Similarly, pressure-to-eat was inversely associated with HRQoL [*β* = −0.438 (95% CI −0.843; −0.033), *p* = 0.034] and demonstrated a significant linear association (*p* = 0.034), whereas the non-linear component was not statistically significant (*p* = 0.097). For monitoring, the linear association was also significant [*β* = 0.470 (95% CI 0.005; 0.936), *p* = 0.048]; however, a significant non-linear component (*p* < 0.001) indicated that the relationship was not purely linear. Specifically, HRQoL decreased with increasing parental monitoring up to approximately 3.5 points on the monitoring scale, after which HRQoL increased at the highest levels of monitoring.

## 4 Discussion

The main finding of this study is that parental feeding practices of restricting or pressuring children to eat were associated with lower HRQoL in children, whereas monitoring showed a non-linear association with HRQoL, with higher HRQoL observed only at the highest levels of monitoring. Similar patterns of association were observed in the age-stratified analyses. However, the sample of children aged <8 years was approximately three times larger than that of children aged ≥8 years, which may have reduced the statistical power to detect associations for restriction and monitoring in the older age group. Coercive control parental feeding practices may be associated with lower children’s HRQoL, potentially through mechanisms such as poorer self-regulation, body dissatisfaction and lower self-esteem. Conversely, structured parental feeding practices may be related to better HRQoL by supporting skills such as self-control, self-care, autonomy and eating self-regulation. Empirical evidence examining the association between parental feeding practices and children’s HRQoL remains limited, making direct comparisons with previous studies difficult.

We hypothesized that coercive control feeding practices, such as restriction and pressure-to-eat, would be associated with lower children’s HRQoL, whereas more structured feeding practices, such as monitoring, would be associated with higher children’s HRQoL. Our findings were broadly consistent with our hypothesis, although they reflect associations based on the same parent/proxy respondent’s reports of both feeding practices and children’s HRQoL. For monitoring, the non-linear pattern suggest that regular (mostly/always) rather than sporadic (rarely/sometimes) monitoring was associated with higher HRQoL, potentially through its association with healthier eating behaviors and better eating self-regulation. In line with these findings, the dose–response analysis showed a non-linear association. HRQoL decreased with increasing monitoring up to a score of approximately 3.5, after which higher monitoring scores were associated with higher HRQoL. Confidence intervals widened at lower monitoring scores, indicating greater uncertainty in this range. Although the observed effect sizes were modest, they are consistent with the multifactorial nature of HRQoL, in which parental feeding practices represent only one of many contributing factors. Therefore, while statistically significant, the practical significance of these associations should be interpreted with appropriate caution.

Surprisingly, in the general linear model analysis, adjustments for additional confounders such as children’s adherence to the Mediterranean diet or physical activity level did not attenuate the magnitude of the associations suggesting a certain robustness or the need for additional factors to understand these associations. In the fully adjusted categorical analysis, differences in HRQoL between the highest and lowest tertiles were −2.1 points for restriction, −1.5 points for pressure-to-eat, and +2.3 points for monitoring, corresponding approximately to 0.15–0.24 SD units. Thus, although statistically significant, the magnitude of the observed differences was modest. Although some studies have reported that adherence to the Mediterranean diet and physical activity were positively associated with HRQoL in children and adolescents (2, 3, 25), this was not reflected in the present analysis. This may be explained by the fact that the children included in these studies tended to be older than in this one. For example, according to Esteban-Gonzalo and colleagues (26), the association between adherence to the Mediterranean diet and children’s HRQoL was not as clear in younger children as in older ones. Older children may have already adopted consistent eating habits, while younger children may still be in a learning phase.

Parental feeding practices have been reported to explain part of the variance in parenting style (9). According to the baseline data of a randomized controlled study (9), monitoring, modeling and responsibility were associated with authoritative parenting, characterized by high demands, but also warmth and acceptance (8). On the other hand, restriction, pressure-to-eat and poor monitoring were associated with authoritarian parenting, characterized by high demands, coldness and lack of acceptance (8, 9). Results from a recent meta-analysis indicated that positive and negative parenting styles were positively and negatively associated with youths’ subjective well-being, respectively (27). Parenting styles reflected in children’s feeding practices may provide a useful framework for understanding differences in children’s HRQoL. Authoritative styles may be associated with better children’s HRQoL by promoting a healthy relationship with food and considering children’s preferences and needs which may foster autonomy, self-control and eating self-regulation (13). On the other hand, authoritarian styles may be related to less favorable HRQoL outcomes due to the adoption of restrictive or pressure-to-eat feeding practices and their negative association with children’s eating self-regulation, body image and self-esteem (13). These parenting styles expressed through feeding practices are also associated with other important behaviors that predict children HRQoL such as physical activity or screen time (28).

The present study has several strengths, including addressing an understudied association between parental feeding practices and children’s HRQoL, as well as its substantial sample size. On the other hand the study shows the following limitations. First, both the exposures and outcomes were assessed using parent-reported questionnaires and are therefore subject to the inherent limitations of self-reported data, such as memory bias, misunderstanding and social desirability. The use of a single informant may also have introduced shared-method variance, perceptual bias, and proxy-related reporting bias, potentially inflating the observed associations (29, 30). A set of basic data was collected from the proxy respondent to evaluate different patterns of responses, however some variables included in our model that could influence proxy responses, such as parental educational level, did not appear to influence the results. Nevertheless, future studies should incorporate multiple informants, such as child self-reports of HRQoL where age-appropriate, and/or observational measures of parental feeding practices to reduce this potential bias. Second, the questionnaire used to assess parental feeding practices captures practices typically associated with authoritative and authoritarian parenting styles but does not allow the identification of neglectful and indulgent parenting styles, which may also influence children’s HRQoL. In addition, broader parenting styles, family functioning, and parental mental health were not assessed, although these factors may influence both parental feeding practices and children’s HRQoL. Future studies incorporating these variables would help clarify the mechanisms underlying the observed associations. Third, the cross-sectional design precludes any conclusions regarding the temporal direction or causality of the observed associations. Although our conceptual framework was based on evidence suggesting that parental feeding practices may influence children’s well-being, it is also possible that parents adapt their feeding practices in response to their child’s HRQoL. Longitudinal studies are therefore needed to clarify the directionality of these associations. Finally, KIDSCREEN-10 was originally developed and validated for children and adolescents aged 8 to 18 years, whereas our cohort included children as young as 3 years. The use of the parent-proxy version reduces concerns related to younger children’s ability to understand questionnaire items, while internal consistency was comparable in children younger than 8 years (Cronbach’s *α* = 0.75) and those aged 8 years or older (Cronbach’s *α* = 0.74). Nevertheless, the validity of HRQoL measurements in younger children remains uncertain, and findings for this age group should therefore be interpreted with caution. Reassuringly, age-stratified analyses (<8 vs. ≥8 years) yielded similar overall patterns of association, although future studies should confirm these findings using HRQoL instruments specifically validated for younger children.

## 5 Conclusion

The findings were consistent with our hypothesis that parental pressure-to-eat and restrictive feeding practices were associated with lower children’s HRQoL, whereas monitoring showed a non-linear association with HRQoL, with higher HRQoL observed only at the highest levels of monitoring. If these associations are confirmed in prospective studies using multiple informants to minimize potential reporting bias, future family-based nutrition interventions could consider the role of structured parental feeding practices, which may contribute to better HRQoL in children. These health promotion efforts should consider parenting styles as an important factor associated with children’s health and well-being.

## Data Availability

The raw data supporting the conclusions of this article will be made available by the authors, without undue reservation.
